# Editorial Note: Re-use of laboratory utensils reduces CO2 equivalent footprint and running costs

**DOI:** 10.1371/journal.pone.0349896

**Published:** 2026-05-28

**Authors:** The PLOS One Editors

The *PLOS One* Editors issue this Editorial Note because this article [1] was identified as one of a series of submissions for which we have concerns about aspects of the peer review process. Readers are encouraged to approach the article [1] with careful consideration.

The Editors have no evidence of any author involvement in the peer review concerns.

## References

[1] Farley M, Nicolet BP. Re-use of laboratory utensils reduces CO2 equivalent footprint and running costs. PLoS One. 2023;18(4):e0283697. doi: 10.1371/journal.pone.0283697 37043455 PMC10096514

